# Neoadjuvant Treatment Strategies for Resectable Proximal Gastric, Gastroesophageal Junction and Distal Esophageal Cancer

**DOI:** 10.3390/cancers14071755

**Published:** 2022-03-30

**Authors:** M. Usman Ahmad, Christopher Javadi, George A. Poultsides

**Affiliations:** Section of Surgical Oncology, Department of Surgery, Stanford University, Stanford, CA 94205, USA; musahmad@stanford.edu (M.U.A.); javadi@stanford.edu (C.J.)

**Keywords:** chemoradiation, neoadjuvant, chemotherapy, radiation, gastroesophageal, adenocarcinoma, surgery, squamous cell carcinoma

## Abstract

**Simple Summary:**

The five-year survival for resectable proximal gastric, gastroesophageal junction (GEJ), and distal esophageal cancer ranges from 30 to 60% globally. Neoadjuvant and/or perioperative therapy has emerged as a treatment tool to improve patient selection for surgery, resectability, and locoregional control of the disease. As a result, treatment strategies have evolved from the first trials in the late 1980s to the pivotal CROSS trial updated in 2015. The review summarizes current clinical trials and treatment recommendations with regard to neoadjuvant and/or perioperative therapy for patients with adenocarcinoma and squamous cell carcinoma of the distal esophagus, GEJ, and proximal stomach.

**Abstract:**

Neoadjuvant treatment strategies for resectable proximal gastric, gastroesophageal junction (GEJ), and distal esophageal cancer have evolved over several decades. Treatment recommendations differ based on histologic type—squamous cell carcinoma (SCC) versus adenocarcinoma (AC)—as well as the exact location of the tumor. Recent and older clinical trials in this area were critically reviewed. Neoadjuvant chemoradiation with concurrent taxane- or fluoropyrimidine-based chemotherapy has an established role for both AC and SCC of the distal esophagus and GEJ. The use of perioperative chemotherapy for gastric AC is based on the FLOT4 and MAGIC trials; however, the utility of neoadjuvant chemoradiation in this setting requires further evaluation. Additional clinical trials evaluating chemotherapy, targeted therapy, immunotherapy, and radiation that are currently in process are highlighted, given the need for further disease control.

## 1. Introduction

Gastroesophageal junction (GEJ) cancer has historically been composed of distinct anatomic locations, including proximal gastric, true GEJ, and distal esophagus, based on the Siewert classification system [1,2]. Globally, esophageal and stomach cancer are common and comprise a significant percentage of new cancer cases (3.2% & 5.7%) and deaths (5.4% & 8.2%) [3].

In the United States (US), the rate of esophageal cancer is 5.7 per 100,000, with up to 50% presenting with localized or regional disease [3,4]. Projections for 2022 include 20,640 new cases and 16,410 deaths in the US [5]. Over time adenocarcinoma (AC) has overtaken squamous cell carcinoma (SCC) as the more common cancer of the esophagus in the US [4]. For all stages combined, the 5-year survival varies slightly by histology for AC (24.2%) and SCC (21.1%) [4]. For all histologies combined, the 5-year survival varies from 46.4% for localized to 25.6% for regional esophageal cancer [6].

Gastric cancer incidence varies geographically, with rates ranging from 3.0 to 32.2 per 100,000 depending on country and gender [3]. Projections for 2022 include 26,380 new cases and 11,090 deaths in the US [7]. The five-year survival of gastric AC varies globally, with rates higher than 60% in Japan and Korea vs. 30–40% in the US and Europe [8,9,10]. Other rarer types of esophageal or gastric cancer include adenosquamous carcinoma, undifferentiated carcinoma, various neuroendocrine cancers, adenocarcinoma with neuroendocrine features, lymphoepithelial carcinoma, parietal cell carcinoma, medullary carcinoma, gastrointestinal stromal tumors, sarcomas, and lymphomas [11,12]. These rare histologies will not be covered by this review.

According to the National Comprehensive Cancer Network (NCCN) Guidelines, distal esophagus and GEJ cancers are managed distinctly from proximal gastric cancer [13]. These anatomic subtypes can be further subdivided based on histopathology: AC vs. SCC. In AC or SCC of the esophagus, pTis to T1a may be managed with endoscopic resection or esophagectomy, while endoscopic resection for T1b disease is controversial [13]. T2 to T4a disease is treated with preoperative chemoradiation, perioperative chemotherapy, or preoperative chemotherapy followed by surgery [13]. Of these therapeutic strategies, preoperative chemoradiation is preferred [13]. Category 1 recommendations (strong recommendation) for chemotherapy include: paclitaxel + carboplatin, fluorouracil + oxaliplatin, or fluorouracil + cisplatin [13]. Trastuzumab should be added for HER2 positive adenocarcinoma [13]. Preoperative radiation is given in a total dose of 41.4–50.4 Gray (Gy) [13].

NCCN guidelines recommend endoscopic resection or surgery for Tis-T1b gastric cancer [14]. Resectable T2+ disease should be offered perioperative chemotherapy (category 1) or preoperative chemoradiation (category 2B) followed by surgery in appropriate candidates [14]. Category 1 recommendations for perioperative chemotherapy include: fluorouracil + leucovorin + oxaliplatin + docetaxel, fluoropyrimidine + oxaliplatin, or fluorouracil + cisplatin [14]. Trastuzumab should be added for HER2 positive adenocarcinoma [14]. Although preoperative chemoradiation is not preferred, doses for radiation range between 45 and 50.4 Gy [14]. Minor differences exist between NCCN and Japanese guidelines, including the role of neoadjuvant chemotherapy and nodal dissection. In Japanese guidelines, both T2-T4, M0 disease, and select M1 disease may be offered neoadjuvant chemotherapy (weak recommendation), D2 resection, and paraaortic nodal dissection (weak recommendation) [15]. Recommended first-line regimens include: S1 + Cisplatin/Oxaliplatin, Capecitabine + Cisplatin/Oxaliplatin, or FOLFOX (Fluorouracil, Leucovorin, and Oxaliplatin) [15]. Trastuzumab is added for HER2 positive adenocarcinoma, and FOLFOX and oxaliplatin are not preferred for this sub-type [15]. Adjuvant chemotherapy should be offered for Stage II/III (excluding T1 or T3N0, no adjuvant chemotherapy) disease with: S1, S1 + docetaxel, or capecitabine + oxaliplatin [15].

This review is focused on neoadjuvant therapies for resectable disease and will highlight the most important clinical trials and meta-analyses.

## 2. Squamous Cell Carcinoma (SCC) of the Distal Esophagus and GEJ

### 2.1. Completed Clinical Trials

#### 2.1.1. Neoadjuvant Chemotherapy vs. Surgery Alone

Six randomized controlled trials (RCTs) evaluated the role of neoadjuvant chemotherapy followed by surgery vs. surgery alone for esophageal SCC [16,17,18,19,20,21,22]. Two trials included both AC and SCC patients [23,24]. Studies are listed in Table 1 and Table 2.

In 1988, Roth et al. compared 39 patients treated either with upfront surgery or with perioperative chemotherapy (Cisplatin + Vindesine + Bleomycin) followed by resection. There were significant differences in median overall survival (mOS) when comparing responders vs. non-responders vs. upfront surgery (20 vs. 6.2 vs. 8.6 months) [16]. However, the benefit of this effect was not seen when strictly analyzed by the treatment arm [16]. Similarly, Schlag (*n* = 46) and Maipang (*n* = 46) conducted RCTs which found no benefit to neoadjuvant chemotherapy vs. surgery alone [17,18]. Law et al. evaluated 147 patients treated with neoadjuvant chemotherapy (Cisplatin + 5-Fluorouracil) followed by resection and found significant differences in mOS when comparing responders vs. surgery alone (42.2 vs. 13.8 months, *p* = 0.003) [19]. There were no significant differences in survival in the trial by Baba et al. (*n* = 42) evaluating the use of Cisplatin + 5-Fluorouracil (5-FU) + Leucovorin [20]. Ancona (*n* = 94) et al. evaluated the use of cisplatin + 5-FU and found significant differences in 5-year survival rate when comparing responders vs. non-responders (60% vs. 12%, *p* = 0.0002) [21]. Boonstra (*n* = 169) et al. evaluated the use cisplatin + etoposide with significant improvement in survival vs. surgery alone (HR 0.71, 95% CI 0.51–0.98, *p* = 0.03) [22]. The OEO2 trial evaluated both AC and SCC (*n* = 247) patients with cisplatin + 5-FU with significant differences in OS across the entire study, although the SCC subgroup failed to reach significance (HR 0.81, 95% CI 0.61–1.07) [23]. Most recently, RTOG 8911 evaluated both AC and SCC (*n* = 207) with the same regimen and found no significant difference in overall survival, though multivariate analysis showed a survival advantage in patients who had a response to chemotherapy, <10% weight loss, and AC histology [24].

In summary, multiple RCTs report differences in OS based on pathologic response to chemotherapy [16,19,21,24]. This effect was seen across multiple regimens without significant changes in OS across study arms and should be studied further. Taken together, these data indicate that only select patients gain benefit from neoadjuvant chemotherapy vs. surgery alone for esophageal SCC.

#### 2.1.2. Neoadjuvant Chemoradiation

Three RCTs compared chemoradiation vs. surgery alone for patients with esophageal SCC [25,26,27]. Five RCTs evaluated chemoradiation vs. surgery or chemotherapy + surgery in a mixed cohort (AC & SCC) [29,30,31,32,34]. Studies are listed in Table 1 and Table 2.

In 1992, Nygaard et al. (*n* = 217) evaluated the independent effects of chemotherapy and radiation (35 Gy) prior to surgical resection. They found no benefit of chemotherapy, while radiation improved OS either with (*p* = 0.05) or without chemotherapy (*p* = 0.01) [25]. There was no difference in either of these groups compared to surgery alone [25]. Similarly, Bosset et al. (*n* = 282) treated patients with cisplatin + 37 Gy and found no differences in survival compared to surgery [26]. Further trials added 5-FU to attempt to improve responses. Lee et al. (*n* = 101) treated patients with cisplatin + 5-FU + 45.6 Gy with no benefit in OS compared to surgery alone [27]. Urba et al. evaluated a mixed group treated with cisplatin + 5-FU + 45 Gy and found that tumor size, SCC histology, and age > 70 were significantly associated with worse survival on multivariate analysis [29]. Burmesiter et al. (*n* = 128) evaluated a mixed group treated with cisplatin + 5-FU + 35 Gy with no significant difference in OS, though there was a higher rate of R0 resection in the chemoradiation group (80% vs. 59%, *p* = 0.002) [30]. FFCD 9901 (*n* = 194) evaluated cisplatin + 5-FU + 45 Gy with no benefit observed in the treatment group [31]. NeoRes I reported a mixed cohort including SCC (*n* = 50) treated with cisplatin + 5-FU with and without 40 Gy radiation and found no survival benefit with the addition of radiation [34]. However, SCC was 2.49 times more likely to have a complete histopathologic response (*p* = 0.049) [34]. Collectively, these studies demonstrated that platinum- and fluoropyrimidine- based regimens with radiation did not improve survival over surgery alone. The pivotal Chemoradiotherapy for Oesophageal Cancer Followed by Surgery Study (CROSS) established the benefit of neoadjuvant taxane-based chemoradiation for >T1 esophageal cancer [32]. The survival benefit was greatest in the SCC subgroup, with mOS of 81.6 months in the neoadjuvant chemoradiotherapy plus surgery group and 21.1 months in the surgery alone group (HR 0.48, 95% CI 0.28–0.83, *p* = 0.003) [32]. The SCC group also had a higher rate of pathologic complete response (49%) compared to the AC group (23%, *p* = 0.008).

Succinctly, although neoadjuvant chemoradiation did not seem to offer a survival benefit when compared to surgery alone in several trials, the CROSS trial was able to establish the benefit of neoadjuvant chemoradiation for esophageal SCC [25,26,27,29,30,31]. Furthermore, although the additive effect of chemotherapy to radiation was inconsistent in prior studies [25,34], the CROSS trial demonstrated a survival benefit compared to surgery when using chemoradiation with the addition of paclitaxel which is fundamentally different from other studies.

#### 2.1.3. Neoadjuvant Chemoradiation with Other Therapies

One RCT evaluated induction chemotherapy prior to chemoradiation in SCC [28]. Another RCT evaluated intratumoral paclitaxel in addition to chemoradiation in a mixed cohort [33]. These studies are listed in Table 1 and Table 2.

Yoon et al. (*n* = 97, includes 2 AC patients) evaluated induction chemotherapy (oxaliplatin + S1) followed by 47 Gy of chemoradiation vs. chemoradiation alone (47 Gy) with no significant difference in overall survival [28]. Dewitt et al. evaluated in a mixed cohort (*n* = 87) the use of intratumoral injection of paclitaxel in conjunction with chemoradiation (cisplatin + 5-FU + 50.4 Gy) compared to chemoradiation alone and found improved pathologic response in the group with chemoradiation alone (26.2% vs. 12.5%, *p* = 0.046) [33].

Based on these studies, there does not appear to be added benefit of induction chemotherapy or intratumoral injection of paclitaxel in addition to neoadjuvant chemoradiation for esophageal SCC.

## 3. Distal Esophagus or GEJ Adenocarcinoma

### 3.1. Completed Clinical Trials

#### 3.1.1. Neoadjuvant Chemotherapy vs. Surgery Alone

One RCT evaluated neoadjuvant chemotherapy vs. surgery in patients with AC of the esophagus or GEJ [35]. Two RCTs evaluated neoadjuvant chemotherapy in a mixed population, including AC and SCC [23,24]. These studies are listed in Table 2 and Table 3.

Ychou et al. (*n* = 224) evaluated perioperative chemotherapy (cisplatin + 5-FU) vs. surgery with improvement in OS (HR 0.69, 95% CI 0.50–0.95, *p* = 0.02) [35]. OEO2 evaluated both AC (*n* = 533) and SCC patients with cisplatin + 5-FU and found a statistically significant overall survival benefit across the entire study population (HR 0.84, 95% CI 0.72–0.98, *p* = 0.03). However, in a subset analysis of the AC subgroup, this difference did not achieve statistical significance (HR 0.86, 95% CI 0.71–1.05, NS) [23]. RTOG 8911 evaluated both AC (*n* = 123) and SCC with the same regimen (cisplatin + 5-FU) with no significant difference seen in OS [24]. However, on multivariate analysis, improved OS was seen with AC histology [24].

In summary, based on the available information specific to distal esophageal or GEJ adenocarcinoma, neoadjuvant cisplatin + 5-FU chemotherapy has demonstrated a survival benefit over surgery alone in some studies, but not others. The combination of fluoropyrimidine- and platinum-based chemotherapy may be beneficial for patients who cannot tolerate triplet chemotherapy regimens or chemoradiation.

#### 3.1.2. Neoadjuvant Chemotherapy Regimens

Five RCTs have compared different perioperative and neoadjuvant chemotherapy regimens for distal esophageal and GEJ AC [36,37,38,39,40]. These studies are listed in Table 3.

FLOT65+ (*n* = 43) evaluated perioperative chemotherapy with 5-FU + Leucovorin + Oxaliplatin +/− Docetaxel (FLO vs. FLOT)) and found a nonsignificant trend towards improved progression free survival (PFS) with the triplet FLOT regimen [36]. MRC ST03 (*n* = 1063) evaluated perioperative chemotherapy (Epirubicin + Cisplatin + Capecitabine +/− Bevacizumab) and found no significant survival benefit and an increased risk of anastomotic leak with bevacizumab [37]. MRC OEO5 (*n* = 897) evaluated neoadjuvant chemotherapy (Cisplatin + 5-FU vs. Epirubicin + Cisplatin + Capecitabine) with no significant differences in OS [38]. FLOT4 (*n* = 716) demonstrated a survival advantage of perioperative 5-FU + Leucovorin + Oxaliplatin + Docetaxel (FLOT) compared with Epirubicin + Cisplatin + 5-FU/Capecitabine (ECF/ECX) with 56% of patients with distal esophageal/GEJ adenocarcinoma (HR 0.77, 95% CI 0.63–0.94, *p* = 0.012) [39]. The RESOLVE trial (*n* = 1022) evaluated perioperative (Oxaliplatin + S1) vs. adjuvant (Oxaliplatin + S1 or Oxaliplatin + Capecitabine) with a benefit in the perioperative treatment group for disease free survival (DFS, HR 0.77, 95% CI 0.61–0.97, *p* = 0.027) [40].

Summarizing the above, perioperative chemotherapy regimens have shown a survival benefit in distal esophageal and GEJ AC. Bevacizumab does not appear to improve survival and may increase complications related to wound healing. FLOT (5-FU + Leucovorin + Oxaliplatin + Docetaxel) improves OS compared to ECF (Epirubicin + Cisplatin + 5-FU/Capecitabine). The FLOT regimen has emerged as the standard of care currently for patients fit enough to receive it. Of note, up to 50% of these studies pooled patients with both distal esophageal/GEJ AC and proximal gastric AC, limiting our ability to draw conclusions on each subgroup.

#### 3.1.3. Neoadjuvant Chemoradiation vs. Surgery Alone

Two RCTs have evaluated neoadjuvant chemoradiation compared to surgery [41,42]. Four RCTs evaluated chemoradiation vs. surgery or chemotherapy + surgery in a mixed cohort (AC & SCC) [29,30,31,32]. Studies are listed in Table 2 and Table 3.

Several trials have evaluated the benefit of neoadjuvant chemoradiation. Zhao et al. showed in a Phase II study of 76 patients that neoadjuvant chemoradiation with Oxaliplatin + Capecitabine + 45 Gy was associated with an increased rate of R0 resection versus surgery alone (100% vs. 80%, *p* < 0.05) [41]. However, there was no survival benefit demonstrated. Using the same regimen, Tian et al. (*n* = 132) evaluated neoadjuvant chemoradiation (Oxaliplatin + Capecitabine + 45 Gy) vs. surgery and found an improvement in 3-year OS (63.4% vs. 52.2%, *p* = 0.019) [42]. Similarly, Urba et al. evaluated a mixed group treated with Cisplatin + 5-FU + 45 Gy, and multivariate analysis showed AC status to be associated with improved OS [29]. However, Burmesiter et al. evaluated a mixed group treated with Cisplatin + 5-FU + 35 Gy with no significant difference in OS, with no AC sub-group analysis performed [30]. In addition, FFCD 9901 evaluated cisplatin + 5-FU + 45 Gy with no benefit overall for AC [31]. Although up to this point there appeared to be some benefit in neoadjuvant chemoradiation using a fluoropyrimidine-based regimen, a major trial with an alternative regimen was soon thereafter completed. The CROSS trial reported a mixed cohort including AC (*n* = 275) treated with Carboplatin + Paclitaxel + 41.4 Gy. Though the greatest benefit of chemoradiation was seen in the SCC subgroup, patients with AC also had significantly improved survival (HR 0.73, 95% CI 0.55–0.98, *p* = 0.01) [32].

In summary, three trials have shown that neoadjuvant chemoradiation improves survival in patients with distal esophageal and GEJ AC [29,32,42]. Cisplatin + 5-FU with radiation appears to improve OS in AC vs. SCC, while Carboplatin + Paclitaxel with radiation is effective for both, with a greater effect on SCC than AC. The interaction of chemotherapy and histology requires further evaluation. Overall, neoadjuvant chemoradiation offers a benefit in OS when compared to surgery alone for AC of the distal esophagus or GEJ.

#### 3.1.4. Neoadjuvant Chemoradiation vs. other Therapy

Seven RCTs evaluated neoadjuvant chemoradiation compared to induction chemotherapy and chemoradiation [34,43,44,45,46,47,48]. One RCT evaluated intratumoral paclitaxel in addition to chemoradiation in a mixed cohort [33]. Studies are listed in Table 2 and Table 3.

Several trials have evaluated the benefit of induction chemotherapy. Stahl et al. evaluated neoadjuvant chemoradiation with induction chemotherapy (5-FU + Leucovorin + Cisplatin + 30 Gy) vs. chemotherapy with no significant difference in 3-year survival possibly due to sample size (*n* = 76, 47.2% vs. 27.7%, *p* = 0.07) [43]. Ajani et al. (*n* = 126) evaluated neoadjuvant chemoradiation with induction chemotherapy (Oxaliplatin + 5-FU + 50.4 Gy) vs. neoadjuvant chemoradiation alone and found no significant change in OS [45]. The POET trial (*n* = 65, including 4 SCC) evaluated neoadjuvant chemoradiation with induction chemotherapy (5-FU + Leucovorin + Cisplatin & Etoposide + Cisplatin + 30 Gy) vs. chemotherapy alone (5-FU + Leucovorin + Cisplatin), with a significant improvement in PFS (HR 0.37, 95% CI 0.16–0.85, *p* = 0.01), and an improvement in OS that approached statistical significane (HR 0.65, 95% CI 0.42–1.01, *p* = 0.055) [46]. Other trials have evaluated the benefit of radiation with a given chemotherapy regimen. Burmeister (*n* = 75) et al. evaluated neoadjuvant chemoradiation (Cisplatin + 5-FU + 35 Gy) vs. chemotherapy (Cisplatin + 5-FU) with improvement in pathologic response but no significant differences in PFS or OS [44]. Similarly, NeoRes I studied a mixed cohort including AC (*n* = 131) treated with cisplatin + 5-FU +/− 40 Gy and reported no significant difference in survival [34]. Combining the two aforementioned strategies, the AGITG DOCTOR trial (*n* = 66) evaluated neoadjuvant chemotherapy (Cisplatin + 5-FU + Docetaxel) with or without chemoradiation (45 Gy) after non-response to initial regimen documented by Positron Emission Tomography (PET). DCF + radiation vs. DCF alone resulted in a non-significant difference in 5-year OS to 46% vs. 31% [47]. This improvement in the 5-year survival rate was clinically comparable to the 53% 5-year survival of responders [47]. Although clinically significant, the study was underpowered for statistical significance [47]. Interestingly, NeoSCOPE (*n* = 85) evaluated two neoadjuvant chemoradiation regimens (Oxaliplatin + Capecitabine + 45 Gy vs. Carboplatin + Paclitaxel + 45 Gy) with improved OS in the latter treatment group (HR 0.48, 95% CI 0.24–0.95, *p* = 0.035) [48]. Lastly, Dewitt et al. evaluated a mixed cohort for treatment with intratumoral paclitaxel in conjunction with chemoradiation (cisplatin + 5-FU + 50.4 Gy) compared to chemoradiation with no improvement in OS [33].

In summary, the first two trials approached significance demonstrating the benefit in adding neoadjuvant chemoradiation after induction chemotherapy compared to the same induction chemotherapy regimen [43,46]. There was no benefit of induction chemotherapy prior to chemoradiation vs. chemoradiation alone in a single trial [45]. Two trials did not show a benefit of chemoradiation compared to chemotherapy alone [34,44]. However, the AGITG DOCTOR trial showed that for patients not responding to initial chemotherapy, offering second-line chemotherapy and radiation improved survival [47]. This finding may support “adaptive” chemoradiation as a treatment strategy in the event of no response to induction chemotherapy. Finally, there appears to be more benefit with chemoradiotherapy with taxol vs. fluoropyrimidine-based chemotherapy based on the NeoSCOPE trial.

## 4. Gastric AC

### 4.1. Completed Clinical Trials

#### 4.1.1. Neoadjuvant Chemotherapy vs. Surgery Alone

Three RCTs evaluated neoadjuvant or perioperative chemotherapy vs. surgery alone in gastric AC [49,50,51]. One trial evaluated a mixed population, including both gastric and GEJ cancer [35]. Studies are listed in Table 4.

Two trials evaluated the use of perioperative chemotherapy. The MAGIC (Medical Research Council Adjuvant Gastric Infusional Chemotherapy) trial established chemotherapy as the standard of care for patients with resectable gastric AC [50]. Overall, 74% of patients had gastric AC (*n* = 372) and received perioperative chemotherapy (epirubicin, cisplatin, and 5-FU) vs. surgery alone with improved overall survival (HR 0.75, 95% CI 0.60–0.93, *p* = 0.009) [50]. The French FNCLCC/FFCD trial by Ychou et al. had similar results. 224 patients with GEJ (75%) or gastric (25%) adenocarcinoma were randomized to perioperative chemotherapy with Cisplatin + 5-FU vs. surgery alone with improvement in OS (HR 0.69, 95% CI 0.50–0.95, *p* = 0.02) [35]. Two other trials evaluated a regimen with neoadjuvant chemotherapy alone. Wang et al. (*n* = 60) evaluated neoadjuvant capecitabine vs. surgery with no significant change in 5-year OS (40% vs. 23%, *p* = 0.17), likely because the study was underpowered to show an effect of single-agent therapy [49]. Lastly, the CRT 40954 (*n* = 144) evaluated neoadjuvant chemotherapy (Cisplatin + Leucovorin + 5-FU) vs. surgery and failed to show significant survival benefit (mOS 64.6 vs. 52.2 M, *p* = 0.466), although more patients had R0 resection in the neoadjuvant group (81.9% vs. 66.7%, *p* = 0.036) [51].

Based on the above, trials with perioperative (rather than neoadjuvant only) chemotherapy appeared to show more benefit with the caveat that the FFCD trial included a minority of gastric AC patients. Perioperative chemotherapy may be offered in patients with resectable gastric AC greater than the cT1N0 stage based on the results of the MAGIC trial.

#### 4.1.2. Neoadjuvant Chemotherapy Regimen

Three trials compared different neoadjuvant chemotherapy regimens prior to surgery [36,37,40] for gastric AC. One trial included a mixed population with both GEJ and gastric cancer [39]. Studies are listed in Table 4.

**Figure 1:**
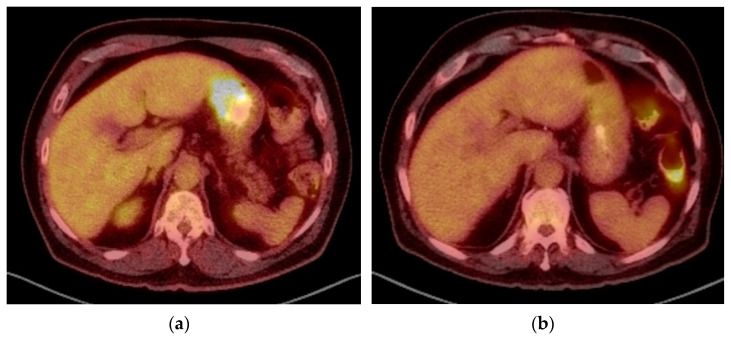
Patient at presentation (**a**) and after 4 cycles of FLOT (**b**) showing response to treatment based on PET scan.

All of the following trials evaluated perioperative chemotherapy in various regimens. FLOT65+ (*n* = 21 gastric AC of a total of 43 patients) evaluated perioperative chemotherapy (5-FU + Leucovorin + Oxaliplatin +/− Docetaxel) with a no significant differences identified [36]. FLOT4 (*n* = 716) evaluated perioperative chemotherapy with 5-FU + Leucovorin + Oxaliplatin + Docetaxel (FLOT) vs. Epirubicin + Cisplatin + 5-FU/Capecitabine (ECF) with improved OS in the FLOT group (HR 0.77, 95% CI 0.63–0.94, *p* = 0.012) [39]. However, only 44% of these patients had gastric cancer, reducing the specificity of these findings. Positron emission tomography (PET) of a patient treated with four cycles of preoperative FLOT with significant radiographic/metabolic response is shown in Figure 1. MRC ST03 (*n* = 383 of 1063 gastric AC) evaluated perioperative chemotherapy with Epirubicin + Cisplatin + Capecitabine +/− Bevacizumab with no significant differences in OS. However, there was an additional risk of anastomotic leak with bevacizumab [37]. The RESOLVE trial (*n* = 649 gastric AC of a total of 1022 patients) evaluated perioperative (Oxaliplatin + S1) vs. adjuvant (Oxaliplatin + S1 or Oxaliplatin + Capecitabine) with a benefit in the perioperative treatment group (HR 0.66, 95% CI 0.61–0.97, *p* = 0.028).

Taken together, these data indicate that FLOT is the perioperative regimen of choice for younger patients with gastric AC and that perioperative chemotherapy offers survival benefits compared to adjuvant chemotherapy alone.

#### 4.1.3. Neoadjuvant Chemoradiation

One RCT evaluated chemoradiation in patients with gastric AC [52]. One trial with a mixed population including gastric and GEJ cancer was included [43]. Studies are listed in Table 4.

CRITICS (*n* = 653 gastric AC of a total of 788 patients) evaluated neoadjuvant chemotherapy with adjuvant chemoradiation (Epirubicin + Cisplatin/Oxaliplatin + Capecitabine and Cisplatin + Capecitabine + 45 Gy) vs. perioperative chemotherapy, with no significant improvement in OS [52]. Stahl et al. evaluated (*n* = 18 of 94 with gastric resection) neoadjuvant chemoradiation with induction chemotherapy (5-FU + Cisplatin + Leucovorin + 30 Gy) vs. neoadjuvant chemotherapy with no difference in 3-year survival (47.2% vs. 27.7%, *p* = 0.07) [43]. However, this trial has a smaller proportion of patients with gastric cancer.

Based on the above, the role of neoadjuvant chemoradiation is unclear for patients with gastric AC.

## 5. Meta-Analyses

### 5.1. Distal Esophageal and GEJ ACC and SC

The value of neoadjuvant chemotherapy vs. surgery alone was assessed by Faron et al. in meta-analysis with significant benefit in OS favoring neoadjuvant chemotherapy (HR 0.83, 95% CI 0.72–0.96, *p* < 0.0001) [53]. There appears to be greater benefit in AC vs. SCC and GEJ vs. esophagus [53]. Kumar et al. were not able to replicate these findings in another meta-analysis; however, they did demonstrate that neoadjuvant chemoradiation conferred significant benefit in OS vs. surgery alone at 3-year survival (OR 0.68, CI 0.52–0.90, *p* = 0.007) [54]. Neoadjuvant chemoradiation vs. chemotherapy was evaluated in a large metanalysis of 18,260 patients with GEJ adenocarcinoma, which found no significant difference in OS, though there was an improvement in RFS (HR 0.85, 95% CI 0.75–0.97, *p* = 0.01) [55]. However, another meta-analysis (*n* = 866) only evaluating clinical trials found a benefit in OS when comparing neoadjuvant chemoradiation to chemotherapy (RR 0.69, 95% CI 0.50–0.96, *p* = 0.03) [56]. Last, a third meta-analysis (*n* = 709) evaluating neoadjuvant chemoradiation vs. chemotherapy for SCC and AC showed a benefit for OS in SCC (RR 1.31, 95% CI 1.10–1.58, *p* = 0.003), but not AC patients [57].

### 5.2. Gastric AC

A meta-analysis of nine RCTs published from 1995 to 2010 assessing the benefit of neoadjuvant chemotherapy vs. surgery alone (*n* = 1056) showed a significantly higher rate of negative lymph node pathology (RR 1.92, 95% CI 1.20–3.06, *p* = 0.006). However, this did not translate into longer OS [58]. The authors postulated that response rates to neoadjuvant chemotherapy might be an influential factor, considering the rate of chemotherapy-related adverse events was 18%. Similarly, an analysis of 6 RCTs (*n* = 781) found no benefit in OS, R0 resection, or postoperative complications with neoadjuvant chemotherapy vs. surgery alone [59]. Neoadjuvant or adjuvant radiation vs. surgery was evaluated in a metanalysis of 9 RCTs (*n* = 832). Neoadjuvant radiation improved overall survival (OR 0.62, 95% CI 0.46–0.84, *p* = 0.002) [60]. Perioperative chemotherapy appears to offer a benefit in survival and may reduce the risk of distant disease after surgical resection (HR 0.48, 95% CI 0.35–0.67, *p* < 0.001) [61].

## 6. Future Directions: Active Clinical Trials

Twenty-six active clinical trials were found, as described in Table 5. Planned or active RCTs are evaluating patients with AC (11), SCC (11), mixed (2), or AC/HER2+ (2) histology.

The majority of these trials (*n* = 16) are evaluating PD-1 (Programmed Death Cell Protein 1) inhibition in combination with radiation, tyrosine kinase inhibitor, HER2 inhibitor, CTLA-4 inhibitor, and fluoropyrimidine +/− taxane-based chemotherapy. PD-L1 expression varies by tumor type, with: 41% in Esophagus/GEJ SCC, 44–52% in Esophagus/GEJ AC, and 23% in Gastric AC (PD-1 or PD-L1) [63,64,65,66]. Thus, unless carefully targeted, patients may not benefit from PD-1 inhibition in these cancer types. In other cancer types, it was previously unclear why patients with low or high PD-L1 expression may respond paradoxically to checkpoint inhibitors. However, a small study in a heterogeneous population of cancer patients showed that PET PD-L1 signal was significantly correlated with response to checkpoint inhibitor but not immunohistochemistry due to significant tumor heterogeneity found on imaging [67]. Thus, future studies may require pre-therapy PET imaging to target patients appropriately. At the time of publication, none of the on-going trials have results available. Furthermore, unfortunately, none of the studies use PD-L1 status to determine patient enrollment for trial design. Thus, although promising if targeted, a broad-based strategy in the current trials will likely produce conflicting results influenced by sample size and overtreatment for patients that may not benefit from the therapy due to lack of PD-L1 at the cost of drug-related adverse events.

One trial is designed to evaluate the benefit of HER2 inhibition with fluoropyrimidine chemotherapy. This study requires HER2 overexpression as enrollment criteria. However, similar critiques of varying levels of HER2 expression, sampling bias, and heterogeneity of tumors have been made of HER2 similarly to PD-L1 [68]. Thus, a future trial may benefit from a radiologically defined patient with PET prior to initiation of therapy similar to a small series of breast cancer patients [69].

Seven trials are designed to evaluate various neoadjuvant chemotherapy or chemoradiation combinations to assess the following: radiation or dosage of radiation, taxane vs. fluoropyrimidine-based regimens, and benefit vs. surgery alone, amongst other aims. In SCC, trials have been designed to evaluate various combinations of treatment. Although previously evaluated, a trial will once again evaluate fluoropyrimidine-based chemoradiation vs. surgery alone. Another trial will evaluate if the benefit to survival in the CROSS trial regimen was based on the chemotherapy or the combination of chemoradiation. Further evaluating radiation, another trial will alter the dose of radiation used in the CROSS trial. Finally, another study evaluated platinum agents vs. fluoropyrimidine chemotherapy with taxane-based chemoradiation. Based on recently published results of this trial, there does not appear to be any difference in survival when comparing regimens offering the possibility of fluoropyrimidine- and taxane-based chemoradiation of patients with SCC [70]. A future trial may extend this to AC patients that are known to have a higher sensitivity to fluoropyrimidine-based regimens. Trials evaluating both AC and SCC are designed to evaluate the optimal chemotherapy regimen. One trial is evaluating FOLFOX vs. carboplatin and paclitaxel. Another is designed to evaluate the CROSS regimen vs. fluoropyrimidine-based chemotherapy. Finally, a trial is evaluating AC alone, comparing a CROSS regimen to fluoropyrimidine-based chemotherapy.

There are also trials that are underway utilizing therapies or techniques used in other cancer types with varying success. One trial is evaluating antiangiogenic therapy in conjunction with neoadjuvant chemotherapy. However, a previous trial using antiangiogenic therapy (bevacizumab) showed increased rates of surgical complications. Finally, a single trial is designed to evaluate the effect of neoadjuvant hyperthermic intraperitoneal chemotherapy (HIPEC). Although frequently used in the metastatic setting, the utility of this technique on resectable disease remains to be seen. The planned clinical trials will help clarify the optimal regimen in conjunction with various adjuvants, including immunotherapy, checkpoint inhibitors, targeted therapies, or intraperitoneal chemotherapy, which may be beneficial for survival in the future.

## 7. Discussion

Substantial progress has been made by adding neoadjuvant and perioperative therapies in various combinations in esophageal, GEJ, and gastric cancers amenable to resection. Neoadjuvant therapy has several potential advantages, including the opportunity to test a tumor’s response to a particular therapeutic regimen and tailor adjuvant therapy based on this response. Neoadjuvant therapy also has the potential to improve R0 resection rates and to improve compliance with systemic therapy. Perhaps most importantly, it provides a window to assess tumor biology, providing insight into the benefit of further medical and surgical intervention for a given patient.

However, more progress is needed given the substantial rate of distant recurrence or metastases. Locoregional control may continue to be improved with patient selection and neoadjuvant and adjuvant treatment strategies. SCC of the esophagus seems to derive greater benefit from the addition of neoadjuvant radiation compared to AC. The benefit of neoadjuvant chemotherapy is clear across both esophageal AC and SCC; however, the optimal regimen is not clear. There is greater chemosensitivity to taxane-based regimens overall; however, the derived benefit individually for SCC vs. AC is not clear. Meanwhile, there appears to be greater chemosensitivity to fluoropyrimidine regimens with AC of the esophagus. This finding was used in the design of the AGITG DOCTOR trial, in which patients not responding to fluoropyrimidine treatment were offered a taxane-based regimen with and without radiation. The addition of radiation to a taxane-based regimen resulted in similar survival to the group of patients that responded to the initial fluoropyrimidine regimen, although the result was not statistically significant based on the study size. This tailored strategy may improve the outcomes of survival for patients with AC of the esophagus resulting in lower rates of local recurrence.

The treatment strategy for gastric AC is centered around perioperative chemotherapy based on the results of the MAGIC and FLOT4 trials. There might be a role for neoadjuvant radiation based on the effect seen in the aforementioned meta-analysis. The impact of extended lymph node resection is controversial and may also impact survival independent of the effects of chemoradiation [71].

Other treatment strategies that need continued exploration are immunotherapy, checkpoint inhibitors, and targeted therapy. Clinical trials are in progress assessing these in the neoadjuvant setting. Currently treated targets include EFGR, HER2, and PD-L1 [72,73,74]. Anti-angiogenic therapy with bevacizumab added additional morbidity to surgery without proven benefit.

## 8. Conclusions

In summary, there continues to be substantial progress in the neoadjuvant and perioperative treatment of distal esophageal, GEJ, and gastric cancers amenable to surgical resection. Selective addition of neoadjuvant chemotherapy and/or radiation is beneficial in specific anatomic and histopathologic subtypes. Adaptive chemoradiation may be a useful protocol using current treatment regimens. Clinical trials will provide further information on the value of immunotherapy or targeted therapies, including HER2, EFGR, PD-L1, and anti-angiogenic therapy. A distant metastatic pattern of failure remains an issue despite locoregional control, and systemic therapy will need further refinement to achieve longer survival post-surgery.

## Figures and Tables

**Table 1:** Randomized Controlled Trials (RCTs) Evaluating Esophageal Squamous Cell Carcinoma (SCC).

Author	Year	Name	Type	Timing	*n*	C	R (Gy)	OS M	Other	Metric	*p*-Value
Neoadjuvant Chemotherapy vs. Surgery Alone											
Roth et al. [16]	1988	-	RCT	Pre + Post	19	Ci + B + Vd	-	9	-	-	NS
				-	20	-	-	9	-	-	
Schlag et al. [17]	1992	-	Phase III	Pre	22	F + Ci	-	10	-	-	NS
				-	24	-	-	10	-	-	
Maipang et al. [18]	1994	-	Phase III	Pre	24	Ci + B + Vb	-	-	31%	3 Y OS	*p* = 0.186
				-	22	-	-	-	36%		
Law et al. [19]	1997	-	RCT	Pre	74	F + Ci	-	16.2	-	-	*p* = 0.4
				-	73	-	-	13.8	-	-	
Baba et al. [20]	2000	-	Phase III	Pre	21	F + L + Ci	-	34.1	-	-	NS
				-	21	-	-	41	-	-	
Ancona et al. [21]	2001	-	Phase III	Pre	47	F + Ci	-	25	-	-	NS
				-	47	-	-	24	-	-	
Boonstra et al. [22]	2011	-	Phase II	Pre	85	Ci + Et	-	-	0.71	HR OS	*p* = 0.03
				-	84	-	-	-			
Neoadjuvant Chemoradiation											
Nygaard et al. [25]	1992	-	RCT	Pre	53	Ci + B	35	-	17%	3 Y OS	1 vs. 2, *p* = 0.05; 2 vs. 3, *p* = 0.01; 4 vs. 3/1, *p* = 0.08/0.3
					56	Ci + B	-	-	3%		
				-	58	-	35	-	21%		
					50	-	-	-	9%		
Bosset et al. [26]	1997	-	RCT	Pre	143	Ci	37	18.6	1.00	HR OS	NS, *p* = 0.78
				-	139	-	-	18.6			
Lee et al. [27]	2004	-	Phase III	Pre	51	F + Ci	45.6	28.2	0.88	HR OS	NS, *p* = 0.69
				-	50	-	-	27.3			
Yoon et al. [28]	2015	-	Phase II	Pre	47	S1 + O × 2	47	-	61%	2 Y OS	NS
					50	S1 + O	50	-	64%		

*n* = Sample Size; C = Chemotherapy; R (Gy) = Radiation Gray; OS M = Overall Survival in Months; RCT = Randomized Controlled Trial; F = 5-FU or 5-Fluorouracil; L = Leucovorin; Ci = Cisplatin; Co = Carboplatin; T = Paclitaxel; T2 = Docetaxel; B = Bleomycin; Et = Etoposide; Vb = Vinblastine; Vd = Vindesine; O = Oxaliplatin; Ep = Epirubicin; Ce = Capecitabine; Bb = Bevacizumab; FO = Oral Fluorouracil; Int = Intratumoral Injection; 1 Y OS = 1 Year Overall Survival; 2 Y OS = 2 Year Overall Survival; 3 Y OS = 3 Year Overall Survival; 5 Y OS = 5 Year Overall Survival; HR DFS = Hazard Ratio Disease Free Survival; HR OS = Hazard Ratio Overall Survival; HR PFS = Hazard Ratio Progression Free Survival; R0 = Microscopic Margin Free Resection; NS = No Significance/Value Not Reported.

**Table 2:** Randomized Controlled Trials (RCTs) Evaluating Esophageal Squamous Cell Carcinoma (SCC) and Adenocarcinoma (AC).

Author	Year	Name	Type	Timing	*n*	C	R (Gy)	OS M	Other	Metric	*p*-Value
Neoadjuvant Chemotherapy vs. Surgery Alone											
Kelsen et al. [24]	2007	RTOG 8911	Phase II	Pre	233	F + Ci	-	14.9	-	-	*p* = 0.53
				-	234	-	-	16.1	-	-	
Allum et al. [23]	2009	OEO2	Phase II	Pre	400	F + Ci	-	-	0.83	HR OS	*p* = 0.04
				-	402	-	-	-			
Neoadjuvant Chemoradiation											
Urba et al. [29]	2001	-	RCT	Pre	50	F + Ci	45	16.9	0.73	HR OS	NS; NS
				-	50	-	-	17.6			
Burmeister et al. [30]	2005	-	Phase III	Pre	128	F + Ci	35	22.2	-	-	NS
				-	128	-	-	19.3	-	-	
Mariette et al. [31]	2014	FFCD 9901	Phase III	Pre	98	F + Ci	45	-	0.99	HR OS	*p* = 0.94
				-	97	-	-	-			
Shapiro et al. [32]	2015	CROSS	Phase III	Pre	180	Co + T	41.4	-	0.66	HR OS	*p* = 0.003
				-	183	-	-	-			
DeWitt et al. [33]	2017	-	Phase IIb	Pre	72	F + Ci + Int T	50.4	-	68%	1 Y OS	*p* = 0.41
				-	65	F + Ci	50.4	-	69%		
von Döbeln et al. [34]	2019	NeoRes I	Phase II	Pre	90	F + Ci	40	31.4	-	-	NS
				-	91	F + Ci	-	36	-	-	

*n* = Sample Size; C = Chemotherapy; R (Gy) = Radiation Gray; OS M = Overall Survival in Months; RCT = Randomized Controlled Trial; F = 5-FU or 5-Fluorouracil; L = Leucovorin; Ci = Cisplatin; Co = Carboplatin; T = Paclitaxel; T2 = Docetaxel; B = Bleomycin; Et = Etoposide; Vb = Vinblastine; Vd = Vindesine; O = Oxaliplatin; Ep = Epirubicin; Ce = Capecitabine; Bb = Bevacizumab; FO = Oral Fluorouracil; Int = Intratumoral Injection; 1 Y OS = 1 Year Overall Survival; 2 Y OS = 2 Year Overall Survival; 3 Y OS = 3 Year Overall Survival; 5 Y OS = 5 Year Overall Survival; HR DFS = Hazard Ratio Disease Free Survival; HR OS = Hazard Ratio Overall Survival; HR PFS = Hazard Ratio Progression Free Survival; R0 = Microscopic Margin Free Resection; NS = No Significance/Value Not Reported.

**Table 3:** Randomized Controlled Trials (RCTs) Evaluating Esophageal and Gastroesophageal Junction (GEJ) Adenocarcinoma (AC).

Author	Year	Name	Type	Timing	*n*	C	R (Gy)	OS M	Other	Metric	*p*-Value
Neoadjuvant Chemotherapy vs. Surgery Alone											
Ychou et al. [35]	2011	-	Phase III	Pre + Post	113	F + Ci	-	-	0.69	HR OS	*p* = 0.02
				-	111	-	-	-			
Neoadjuvant Chemotherapy											
Lorenzen et al. [36]	2013	FLOT 65+	Phase II	Pre + Post	21	F + L + O + T2	-	-	2.02	HR PFS	*p* = 0.09
					22	F + L + O	-	-			
Cunningham et al. [37]	2017	MRC ST03	Phase II/III	Pre + Post	530	Ce + Ci + Ep + Bb	-	-	1.08	HR OS	*p* = 0.36
					533	Ce + Ci + Ep	-	-			
Alderson et al. [38]	2017	MRC OE05	Phase III	Pre	451	F + Ci	-	-	0.90	HR OS	*p* = 0.19
					446	Ce + Ci + Ep	-	-			
Al-Batran et al. [39]	2019	FLOT4	Phase II/III	Pre + Post	360	F/Ce + Ci + Ep	-	35	0.77	HR OS	NS, *p* = 0.012
					356	F + L + O + T2	-	50			
Zhang et al. [40]	2021	RESOLVE	Phase III	Post	345	Ce + O	-	-	0.86	HR DFS	*p* = 0.170
				Post	340	S1 + O	-	-			
				Pre + Post	337	S1 + O	-	-	0.77	HR DFS	*p* = 0.027
Neoadjuvant Chemoradiation vs. Surgery Alone											
Zhao et al. [41]	2015	-	Phase II	Pre	36	Ce + O	45	-	100%	R0	*p* = 0.045
				-	40	-	-	-	80%		
Tian et al. [42]	2021	-	Phase II	Pre	63	Ce + O	45	-	63%	3 Y OS	*p* = 0.019
				-	69	-	-	-	52%		
Neoadjuvant Chemoradiation											
Stahl et al. [43]	2009	-	Phase III	Pre	45	F + L + Ci × 2	30	33.1	-	-	NS
					49	F + L + Ci	-	21.1	-	-	
Burmeister et al. [44]	2011	-	Phase II	Pre	39	F + Ci	35	32	-	-	*p* = 0.83
					36	F + Ci	-	29	-	-	
Ajani et al. [45]	2013	-	Phase II	Pre	63	F + O x 2	50.4	43.7	-	-	*p* = 0.69
					63	F + O	50.4	45.6	-	-	
Stahl et al. [46]	2017	POET	Phase III	Pre	33	F + L + Ci & Ci + Et	30	30.8	0.65	HR OS	NS, *p* = 0.055
					32	F + L + Ci	-	21.1			
Barbour et al. [47]	2020	AGITG DOCTOR	Phase II	Pre	35	F + Ci + T2	45	35	-	-	NS
					31	F + Ci + T2	-	30	-	-	
Mukherjee et al. [48]	2021	NeoSCOPE	Phase II	Pre	42	Ce + O	45	-	0.48	HR OS	*p* = 0.035
					43	Co + T	45	-			

*n* = Sample Size; C = Chemotherapy; R (Gy) = Radiation Gray; OS M = Overall Survival in Months; RCT = Randomized Controlled Trial; F = 5-FU or 5-Fluorouracil; L = Leucovorin; Ci = Cisplatin; Co = Carboplatin; T = Paclitaxel; T2 = Docetaxel; B = Bleomycin; Et = Etoposide; Vb = Vinblastine; Vd = Vindesine; O = Oxaliplatin; Ep = Epirubicin; Ce = Capecitabine; Bb = Bevacizumab; FO = Oral Fluorouracil; Int = Intratumoral Injection; 1 Y OS = 1 Year Overall Survival; 2 Y OS = 2 Year Overall Survival; 3 Y OS = 3 Year Overall Survival; 5 Y OS = 5 Year Overall Survival; HR DFS = Hazard Ratio Disease Free Survival; HR OS = Hazard Ratio Overall Survival; HR PFS = Hazard Ratio Progression Free Survival; R0 = Microscopic Margin Free Resection; NS = No Significance/Value Not Reported.

**Table 4:** Randomized Controlled Trials (RCTs) Evaluating Gastric Adenocarcinoma (AC).

Author	Year	Name	Type	Timing	*n*	C	R (Gy)	OS M	Other	Metric	*p*-Value
Neoadjuvant Chemotherapy vs. Surgery Alone											
Wang et al. [49]	2000	-	RCT	Pre	30	FO	-	-	40%	5 Y OS	*p* = 0.17
				-	30	-	-	-	23%		
Cunningham et al. [50]	2006	MAGIC	Phase III	Pre + Post	250	F + Ci + Ep	-	-	0.75	HR OS	*p* = 0.009
				-	253	-	-	-			
Schuhmacher et al. [51]	2010	CRT 40954	Phase III	Pre	72	F + L + Ci	-	-	0.84	HR OS	*p* = 0.466
				-	72	-	-	-			
Ychou et al. [35]	2011	-	Phase III	Pre + Post	113	F + Ci	-	-	0.69	HR OS	*p* = 0.02
				-	111	-	-	-			
Neoadjuvant Chemotherapy											
Lorenzen et al. [36]	2013	FLOT 65+	Phase II	Pre + Post	21	F + L + O + T2	-	-	2.02	HR PFS	*p* = 0.09
					22	F + L + O	-	-			
Cunningham et al. [37]	2017	MRC ST03	Phase II/III	Pre + Post	530	Ce + Ci + Ep + Bb	-	-	1.08	HR OS	*p* = 0.36
					533	Ce + Ci + Ep	-	-			
Al-Batran et al. [39]	2019	FLOT4	Phase II/III	Pre + Post	360	F/Ce + Ci + Ep	-	35	0.77	HR OS	NS, *p* = 0.012
					356	F + L + O + T2	-	50			
Zhang et al. [40]	2021	RESOLVE	Phase III	Post	345	Ce + O	-	-	0.86	HR DFS	*p* = 0.170
				Post	340	S1 + O	-	-			
				Pre + Post	337	S1 + O	-	-	0.77	HR DFS	*p* = 0.027
Neoadjuvant Chemoradiation											
Stahl et al. [43]	2009	-	Phase III	Pre	45	F + L + Ci × 2	30	33.1	-	-	NS
					49	F + L + Ci	-	21.1	-	-	
Cats et al. [52]	2018	CRITICS	Phase III	Pre + Post	395	Ce + Ci/O + Ep	45	-	1.01	HR OS	*p* = 0.90
					393	Ce + Ci/O + Ep	-	-			

*n* = Sample Size; C = Chemotherapy; R (Gy) = Radiation Gray; OS M = Overall Survival in Months; RCT = Randomized Controlled Trial; F = 5-FU or 5-Fluorouracil; L = Leucovorin; Ci = Cisplatin; Co = Carboplatin; T = Paclitaxel; T2 = Docetaxel; B = Bleomycin; Et = Etoposide; Vb = Vinblastine; Vd = Vindesine; O = Oxaliplatin; Ep = Epirubicin; Ce = Capecitabine; Bb = Bevacizumab; FO = Oral Fluorouracil; Int = Intratumoral Injection; 1 Y OS = 1 Year Overall Survival; 2 Y OS = 2 Year Overall Survival; 3 Y OS = 3 Year Overall Survival; 5 Y OS = 5 Year Overall Survival; HR DFS = Hazard Ratio Disease Free Survival; HR OS = Hazard Ratio Overall Survival; HR PFS = Hazard Ratio Progression Free Survival; R0 = Microscopic Margin Free Resection; NS = No Significance/Value Not Reported.

**Table 5:** Registered Randomized Controlled Trials (RCTs) [62].

Name	NCT	Country	*n*	Design	Type
KEYNOTE-585	NCT03221426	Global	1007	F + Ce + Ci vs. F + Ce + Ci + Plb vs. FLOT vs. FLOT + Plb	AC
-	NCT04592913	Global	900	FLOT vs. FLOT + Db	AC
-	NCT05149807	China	896	TS-1 + O vs. TS-1 + O + SHR-1701	AC
-	NCT04848753	China	500	Ci + T vs. Ci + T + Tpb	SCC
HCHTOG1903	NCT04138212	China	465	Ci + T vs. Co + T + R	SCC
ESOPEC	NCT02509286	Germany	438	FLOT vs. Co + T + R	AC
KEYSTONE-002	NCT04807673	China	342	Ci + T + R vs. Ci + T + Plb	SCC
-	NCT02459457	China	321	Ci + T + R vs. Co + T + R vs. F + T + R	SCC
DANTE	NCT03421288	Germany	295	FLOT vs. FLOT + Atb	AC
-	NCT03381651	China	290	Co + T + R vs. Co + T + Rl	SCC
-	NCT03604991	US	278	Nb vs. Nb + Ib vs. Co + T + R vs. Co + T + R + Nb	AC
-	NCT04208347	China	258	S1 + O vs. S1 + O + Apb vs. S1 + Apb + Cb	AC
INNOVATION	NCT02205047	Global	220	F/Ce + Ci + Ttb vs. F/Ce + Ci + Ttb + Ptb vs. F/Ce + Ci	AC, HER2+
-	NCT05043688	China	204	Co + T + R vs. Co + T + Cb vs. Co + T + Cb + R	SCC
PREVENT	NCT04447352	Germany	200	FLOT vs. FLOT + HIPEC	AC
CELAEC	NCT02972372	China	196	F + Ci + R vs. Surgery	SCC
RAMSES/FLOT7	NCT02661971	Germany	180	FLOT vs. FLOT + Rb	AC
-	NCT04973306	China	176	Co + T + R vs. Co + T + R + Tzb	SCC
NEORACING	NCT05161572	China	152	S1 + O + Sb vs. S1 + O + Sb + R	AC
KEYNOTE-585—CN	NCT04882241	China	120	FLOT vs. FLOT + Plb vs. FLO vs. FLO vs. Plb	AC
PROTECT	NCT02359968	France	106	FOLFOX vs. Co + T	AC or SCC
-	NCT05007145	China	92	PD-1 I + Ci + T vs. Ci + T + R	SCC
-	NCT01404156	Canada	60	FLOT or ECF/ECX vs. Co + T + R	AC or SCC
-	NCT04568200	China	60	Co + T + R vs. Co + T + R + Db	SCC
-	NCT04661150	China	52	Ce + O + Ttb vs. Ce + O + Ttb + Atb	AC, HER2+
-	NCT04937673	China	40	Ci + nab-T + Cb vs. Ci + T + Cb	SCC

US = United States; F = 5-FU/5-Fluorouracil; Ci = Cisplatin; R = Radiation; PD-1I = Programmed Cell Death Protein-1 Inhibitor; T = Paclitaxel; Co = Carboplatin; FOLFOX = Leocovorin, Fluorouracil, Oxaliplatin; Rl = Radiation low dose; FLOT = Fluorouracil, Leucovorin, Oxaliplatin, Docetaxel; ECF = Epirubicin, Cisplatin, Fluorouracil; ECX = Epirubicin, Cisplatin, Capecitabine; Nb = Nivolumab; Ib = Ipilumumab; Tpb = Toripalimab; Db = Durvalumab; Tzb = Tiselizumab; Cb = Camrelizumab; Plb = Pembrolizumab; nab-T = albumin bound Paclitaxel; TS-1 = Tegufur, Gimeracil, Oteracil; Ce = Capecitabine; Ttb = Trastuzumab; Atb = Atezolizumab; HIPEC = Hyperthermic Intraperitoneal Chemotherapy; Rb = Ramucirumab; O = Oxaliplatin; Apb = Apatinib; FLO = Fluorouracil, Leucovorin, Oxaliplatin; Ptb = Pertuzumab; Sb = Sintilimab; TKI = Tyrosine Kinase Inhibitor; HER2 = Human epidermal growth factor receptor 2; CN = China; AC = Adenocarcinoma; SCC = Squamous Cell Carcinoma.

## References

[1] Mazer L.M., Poultsides G.A. (2019). What Is the Best Operation for Proximal Gastric Cancer and Distal Esophageal Cancer?. Surg. Clin. N. Am..

[2] Siewert J.R., Stein H.J., Feith M. (2006). Adenocarcinoma of the Esophago-Gastric Junction. Scand. J. Surg..

[3] Bray F., Ferlay J., Soerjomataram I., Siegel R.L., Torre L.A., Jemal A. (2018). Global Cancer Statistics 2018: GLOBOCAN Estimates of Incidence and Mortality Worldwide for 36 Cancers in 185 Countries. CA Cancer J. Clin..

[4] He H., Chen N., Hou Y., Wang Z., Zhang Y., Zhang G., Fu J. (2020). Trends in the Incidence and Survival of Patients with Esophageal Cancer: A SEER Database Analysis. Thorac. Cancer.

[5] American Cancer Society Cancer Statistics Center: Esophagus at a Glance. https://cancerstatisticscenter.cancer.org/#!/cancer-site/Esophagus.

[6] National Cancer Institute SEER Cancer Stat Facts: Esophageal Cancer. https://seer.cancer.gov/statfacts/html/esoph.html.

[7] American Cancer Society Cancer Statistics Center: Stomach at a Glance. https://cancerstatisticscenter.cancer.org/#!/cancer-site/Stomach.

[8] Sitarz R., Skierucha M., Mielko J., Offerhaus G.J.A., Maciejewski R., Polkowski W.P. (2018). Gastric Cancer: Epidemiology, Prevention, Classification, and Treatment. Cancer Manag. Res..

[9] Matsuda T., Saika K. (2018). Cancer Burden in Japan Based on the Latest Cancer Statistics: Need for Evidence-Based Cancer Control Programs. Ann. Cancer Epidemiol..

[10] National Cancer Institute SEER Cancer Stat Facts: Stomach Cancer. https://seer.cancer.gov/statfacts/html/stomach.html.

[11] Rice T.W., Kelsen D., Blackstone E.H., Ishwaran H., Patil D.T., Bass A.J., Erasmus J.J., Gerdes H., Hofstetter W.L., Amin M.B., Edge S., Greene F., Byrd D.R., Brookland R.K., Washington M.K., Gershenwald J.E., Compton C.C., Hess K.R., Sullivan D.C. (2017). Esophagus and Esophagogastric Junction. AJCC Cancer Staging System.

[12] Ajani J.A., In H., Sano T., Gaspar L.E., Erasmus J.J., Tang L.H., Washington M.K., Gerdes H., Wittekind C.W., Mansfield P.F., Amin M.B., Edge S., Greene F., Byrd D.R., Brookland R.K., Washington M.K., Gershenwald J.E., Compton C.C., Hess K.R., Sullivan D.C. (2017). Stomach. AJCC Cancer Staging System.

[13] Ajani J.A., D’Amico T.A., Bentrem D.J., Chao J., Collier S., Corvera C., Das P., Denlinger C.S., Enzinger P.C., Enzler T. Esophageal and Esophagogastric Junction Cancers, Version 4.2021, NCCN Clinical Practice Guidelines in Oncology. https://www.nccn.org/professionals/physician_gls/pdf/esophageal.pdf..

[14] Ajani J.A., D’Amico T.A., Bentrem D.J., Cooke D., Corvera C., Das P., Enzinger P.C., Enzler T., Fanta P., Farjah F. Gastric Cancer, Version 5.2021, NCCN Clinical Practice Guidelines in Oncology. https://www.nccn.org/professionals/physician_gls/pdf/gastric.pdf.

[15] Japanese Gastric Cancer Association (2021). Japanese Gastric Cancer Treatment Guidelines 2018 (5th Edition). Gastric Cancer.

[16] Roth J.A., Pass H.I., Flanagan M.M., Graeber G.M., Rosenberg J.C., Steinberg S. (1988). Randomized Clinical Trial of Preoperative and Postoperative Adjuvant Chemotherapy with Cisplatin, Vindesine, and Bleomycin for Carcinoma of the Esophagus. J. Thorac. Cardiovasc. Surg..

[17] Schlag P.M. (1992). Randomized Trial of Preoperative Chemotherapy for Squamous Cell Cancer of the Esophagus. Arch. Surg..

[18] Maipang T., Vasinanukorn P., Petpichetchian C., Chamroonkul S., Geater A., Chansawwaang S., Kuapanich R., Panjapiyakul C., Watanaarepornchai S., Punperk S. (1994). Induction Chemotherapy in the Treatment of Patients with Carcinoma of the Esophagus. J. Surg. Oncol..

[19] Law S., Fok M., Chow S., Chu K., Wong J. (1997). Preoperative Chemotherapy versus Surgical Therapy Alone for Squamous Cell Carcinoma of the Esophagus: A Prospective Randomized Trial. J. Thorac. Cardiovasc. Surg..

[20] Baba M., Natsugoe S., Shimada M., Nakano S., Kusano C., Fukumoto T., Aikou T., Akazawa K. (2000). Prospective Evaluation of Preoperative Chemotherapy in Resectable Squamous Cell Carcinoma of the Thoracic Esophagus. Dis. Esophagus.

[21] Ancona E., Ruol A., Santi S., Merigliano S., Chiarion Sileni V., Koussis H., Zaninotto G., Bonavina L., Peracchia A. (2001). Only Pathologic Complete Response to Neoadjuvant Chemotherapy Improves Significantly the Long Term Survival of Patients with Resectable Esophageal Squamous Cell Carcinoma. Cancer.

[22] Boonstra J.J., Kok T.C., Wijnhoven B.P.L., van Heijl M., van Berge Henegouwen M.I., ten Kate F.J.W., Siersema P.D., Dinjens W.N.M., van Lanschot J.J.B., Tilanus H.W. (2011). Chemotherapy Followed by Surgery versus Surgery Alone in Patients with Resectable Oesophageal Squamous Cell Carcinoma: Long-Term Results of a Randomized Controlled Trial. BMC Cancer.

[23] Allum W.H., Stenning S.P., Bancewicz J., Clark P.I., Langley R.E. (2009). Long-Term Results of a Randomized Trial of Surgery With or Without Preoperative Chemotherapy in Esophageal Cancer. J. Clin. Oncol..

[24] Kelsen D.P., Winter K.A., Gunderson L.L., Mortimer J., Estes N.C., Haller D.G., Ajani J.A., Kocha W., Minsky B.D., Roth J.A. (2007). Long-Term Results of RTOG Trial 8911 (USA Intergroup 113): A Random Assignment Trial Comparison of Chemotherapy Followed by Surgery Compared with Surgery Alone for Esophageal Cancer. J. Clin. Oncol..

[25] Nygaard K., Hagen S., Hansen H.S., Hatlevoll R., Hultborn R., Jakobsen A., Mäntyla M., Modig H., Munck-Wikland E., Rosengren B. (1992). Pre-Operative Radiotherapy Prolongs Survival in Operable Esophageal Carcinoma: A Randomized, Multicenter Study of Pre-Operative Radiotherapy and Chemotherapy. The Second Scandinavian Trial in Esophageal Cancer. World J. Surg..

[26] Bosset J.-F., Gignoux M., Triboulet J.-P., Tiret E., Mantion G., Elias D., Lozach P., Ollier J.-C., Pavy J.-J., Mercier M. (1997). Chemoradiotherapy Followed by Surgery Compared with Surgery Alone in Squamous-Cell Cancer of the Esophagus. N. Engl. J. Med..

[27] Lee J.L., Park S.I., Kim S.B., Jung H.Y., Lee G.H., Kim J.H., Song H.Y., Cho K.J., Kim W.K., Lee J.S. (2004). A Single Institutional Phase III Trial of Preoperative Chemotherapy with Hyperfractionation Radiotherapy plus Surgery versus Surgery Alone for Resectable Esophageal Squamous Cell Carcinoma. Ann. Oncol..

[28] Yoon D.H., Jang G., Kim J.H., Kim Y.-H., Kim J.Y., Kim H.R., Jung H.-Y., Lee G.-H., Song H.Y., Cho K.-J. (2015). Randomized Phase 2 Trial of S1 and Oxaliplatin-Based Chemoradiotherapy With or Without Induction Chemotherapy for Esophageal Cancer. Int. J. Radiat. Oncol..

[29] Urba S.G., Orringer M.B., Turrisi A., Iannettoni M., Forastiere A., Strawderman M. (2001). Randomized Trial of Preoperative Chemoradiation Versus Surgery Alone in Patients With Locoregional Esophageal Carcinoma. J. Clin. Oncol..

[30] Burmeister B., Smithers M., Gebski V., Denham J., Devitt P., Ackland S., Findlay M., Dhillon H., Stockler M., Coates A. (2005). Surgery Alone versus Chemoradiotherapy Followed by Surgery for Resectable Cancer of the Oesophagus: A Randomised Controlled Phase III Trial. Lancet Oncol..

[31] Mariette C., Dahan L., Mornex F., Maillard E., Thomas P.-A., Meunier B., Boige V., Pezet D., Robb W.B., Le Brun-Ly V. (2014). Surgery Alone Versus Chemoradiotherapy Followed by Surgery for Stage I and II Esophageal Cancer: Final Analysis of Randomized Controlled Phase III Trial FFCD 9901. J. Clin. Oncol..

[32] Shapiro J., van Lanschot J.J.B., Hulshof M.C.C.M., van Hagen P., van Berge Henegouwen M.I., Wijnhoven B.P.L., van Laarhoven H.W.M., Nieuwenhuijzen G.A.P., Hospers G.A.P., Bonenkamp J.J. (2015). Neoadjuvant Chemoradiotherapy plus Surgery versus Surgery Alone for Oesophageal or Junctional Cancer (CROSS): Long-Term Results of a Randomised Controlled Trial. Lancet Oncol..

[33] DeWitt J.M., Murthy S.K., Ardhanari R., DuVall G.A., Wallner G., Litka P., Daugherty C., Fowers K. (2017). EUS-Guided Paclitaxel Injection as an Adjunctive Therapy to Systemic Chemotherapy and Concurrent External Beam Radiation before Surgery for Localized or Locoregional Esophageal Cancer: A Multicenter Prospective Randomized Trial. Gastrointest. Endosc..

[34] von Döbeln G.A., Klevebro F., Jacobsen A.-B., Johannessen H.-O., Nielsen N.H., Johnsen G., Hatlevoll I., Glenjen N.I., Friesland S., Lundell L. (2019). Neoadjuvant Chemotherapy versus Neoadjuvant Chemoradiotherapy for Cancer of the Esophagus or Gastroesophageal Junction: Long-Term Results of a Randomized Clinical Trial. Dis. Esophagus.

[35] Ychou M., Boige V., Pignon J.-P., Conroy T., Bouché O., Lebreton G., Ducourtieux M., Bedenne L., Fabre J.-M., Saint-Aubert B. (2011). Perioperative Chemotherapy Compared With Surgery Alone for Resectable Gastroesophageal Adenocarcinoma: An FNCLCC and FFCD Multicenter Phase III Trial. J. Clin. Oncol..

[36] Lorenzen S., Pauligk C., Homann N., Schmalenberg H., Jäger E., Al-Batran S.-E. (2013). Feasibility of Perioperative Chemotherapy with Infusional 5-FU, Leucovorin, and Oxaliplatin with (FLOT) or without (FLO) Docetaxel in Elderly Patients with Locally Advanced Esophagogastric Cancer. Br. J. Cancer.

[37] Cunningham D., Stenning S.P., Smyth E.C., Okines A.F., Allum W.H., Rowley S., Stevenson L., Grabsch H.I., Alderson D., Crosby T. (2017). Peri-Operative Chemotherapy with or without Bevacizumab in Operable Oesophagogastric Adenocarcinoma (UK Medical Research Council ST03): Primary Analysis Results of a Multicentre, Open-Label, Randomised Phase 2–3 Trial. Lancet Oncol..

[38] Alderson D., Cunningham D., Nankivell M., Blazeby J.M., Griffin S.M., Crellin A., Grabsch H.I., Langer R., Pritchard S., Okines A. (2017). Neoadjuvant Cisplatin and Fluorouracil versus Epirubicin, Cisplatin, and Capecitabine Followed by Resection in Patients with Oesophageal Adenocarcinoma (UK MRC OE05): An Open-Label, Randomised Phase 3 Trial. Lancet. Oncol..

[39] Al-Batran S.-E., Homann N., Pauligk C., Goetze T.O., Meiler J., Kasper S., Kopp H.-G., Mayer F., Haag G.M., Luley K. (2019). Perioperative Chemotherapy with Fluorouracil plus Leucovorin, Oxaliplatin, and Docetaxel versus Fluorouracil or Capecitabine plus Cisplatin and Epirubicin for Locally Advanced, Resectable Gastric or Gastro-Oesophageal Junction Adenocarcinoma (FLOT4): A R. Lancet.

[40] Zhang X., Liang H., Li Z., Xue Y., Wang Y., Zhou Z., Yu J., Bu Z., Chen L., Du Y. (2021). Perioperative or Postoperative Adjuvant Oxaliplatin with S-1 versus Adjuvant Oxaliplatin with Capecitabine in Patients with Locally Advanced Gastric or Gastro-Oesophageal Junction Adenocarcinoma Undergoing D2 Gastrectomy (RESOLVE): An Open-Label, Superio. Lancet Oncol..

[41] Zhao Q., Li Y., Wang J., Zhang J., Qiao X., Tan B., Tian Y., Shi G., Xu Q., Li R. (2015). Concurrent Neoadjuvant Chemoradiotherapy for Siewert II and III Adenocarcinoma at Gastroesophageal Junction. Am. J. Med. Sci..

[42] Tian Y., Wang Q., Wang J., Qiao X.Y., Zhang J., Lin Y.C., Li Y., Fan L.Q., Yang P.G., Zhao Q. (2021). Neoadjuvant chemoradiotherapy combined with surgery versus direct surgery in the treatment of Siewert type II and III adenocarcinomas of the esophagogastric junction: Long-term prognostic analysis of a prospective randomized controlled trial. Zhonghua Wei Chang Wai Ke Za Zhi.

[43] Stahl M., Walz M.K., Stuschke M., Lehmann N., Meyer H.-J., Riera-Knorrenschild J., Langer P., Engenhart-Cabillic R., Bitzer M., Königsrainer A. (2009). Phase III Comparison of Preoperative Chemotherapy Compared with Chemoradiotherapy in Patients With Locally Advanced Adenocarcinoma of the Esophagogastric Junction. J. Clin. Oncol..

[44] Burmeister B.H., Thomas J.M., Burmeister E.A., Walpole E.T., Harvey J.A., Thomson D.B., Barbour A.P., Gotley D.C., Smithers B.M. (2011). Is Concurrent Radiation Therapy Required in Patients Receiving Preoperative Chemotherapy for Adenocarcinoma of the Oesophagus? A Randomised Phase II Trial. Eur. J. Cancer.

[45] Ajani J.A., Xiao L., Roth J.A., Hofstetter W.L., Walsh G., Komaki R., Liao Z., Rice D.C., Vaporciyan A.A., Maru D.M. (2013). A Phase II Randomized Trial of Induction Chemotherapy versus No Induction Chemotherapy Followed by Preoperative Chemoradiation in Patients with Esophageal Cancer. Ann. Oncol..

[46] Stahl M., Walz M.K., Riera-Knorrenschild J., Stuschke M., Sandermann A., Bitzer M., Wilke H., Budach W. (2017). Preoperative Chemotherapy versus Chemoradiotherapy in Locally Advanced Adenocarcinomas of the Oesophagogastric Junction (POET): Long-Term Results of a Controlled Randomised Trial. Eur. J. Cancer.

[47] Barbour A.P., Walpole E.T., Mai G.T., Barnes E.H., Watson D.I., Ackland S.P., Martin J.M., Burge M., Finch R., Karapetis C.S. (2020). Preoperative Cisplatin, Fluorouracil, and Docetaxel with or without Radiotherapy after Poor Early Response to Cisplatin and Fluorouracil for Resectable Oesophageal Adenocarcinoma (AGITG DOCTOR): Results from a Multicentre, Randomised Controlled Phase II. Ann. Oncol. Off. J. Eur. Soc. Med. Oncol..

[48] Mukherjee S., Hurt C., Radhakrishna G., Gwynne S., Bateman A., Gollins S., Hawkins M.A., Canham J., Grabsch H.I., Falk S. (2021). Oxaliplatin/Capecitabine or Carboplatin/Paclitaxel-Based Preoperative Chemoradiation for Resectable Oesophageal Adenocarcinoma (NeoSCOPE): Long-Term Results of a Randomised Controlled Trial. Eur. J. Cancer.

[49] Wang X.L., Wu G.X., Zhang M.D., Guo M., Zhang H., Sun X.F. (2000). A Favorable Impact of Preoperative FPLC Chemotherapy on Patients with Gastric Cardia Cancer. Oncol. Rep..

[50] Cunningham D., Allum W.H., Stenning S.P., Thompson J.N., Van de Velde C.J.H., Nicolson M., Scarffe J.H., Lofts F.J., Falk S.J., Iveson T.J. (2006). Perioperative Chemotherapy versus Surgery Alone for Resectable Gastroesophageal Cancer. N. Engl. J. Med..

[51] Schuhmacher C., Gretschel S., Lordick F., Reichardt P., Hohenberger W., Eisenberger C.F., Haag C., Mauer M.E., Hasan B., Welch J. (2010). Neoadjuvant Chemotherapy Compared With Surgery Alone for Locally Advanced Cancer of the Stomach and Cardia: European Organisation for Research and Treatment of Cancer Randomized Trial 40954. J. Clin. Oncol..

[52] Cats A., Jansen E.P.M., van Grieken N.C.T., Sikorska K., Lind P., Nordsmark M., Meershoek-Klein Kranenbarg E., Boot H., Trip A.K., Swellengrebel H.A.M. (2018). Chemotherapy versus Chemoradiotherapy after Surgery and Preoperative Chemotherapy for Resectable Gastric Cancer (CRITICS): An International, Open-Label, Randomised Phase 3 Trial. Lancet Oncol..

[53] Faron M., Cheugoua-Zanetsie A.M., Thirion P., Nankivell M., Winter K., Cunningham D., Van der Gaast A., Law S., Langley R., de Vathaire F. (2021). Individual Patient Data Meta-Analysis of Neoadjuvant Chemotherapy Followed by Surgery versus Upfront Surgery for Carcinoma of the Oesophagus or the Gastro-Oesophageal Junction. Eur. J. Cancer.

[54] Kumar T., Pai E., Singh R., Francis N.J., Pandey M. (2020). Neoadjuvant Strategies in Resectable Carcinoma Esophagus: A Meta-Analysis of Randomized Trials. World J. Surg. Oncol..

[55] Petrelli F., Ghidini M., Barni S., Sgroi G., Passalacqua R., Tomasello G. (2019). Neoadjuvant Chemoradiotherapy or Chemotherapy for Gastroesophageal Junction Adenocarcinoma: A Systematic Review and Meta-Analysis. Gastric Cancer.

[56] Zhao X., Ren Y., Hu Y., Cui N., Wang X., Cui Y. (2018). Neoadjuvant Chemotherapy versus Neoadjuvant Chemoradiotherapy for Cancer of the Esophagus or the Gastroesophageal Junction: A Meta-Analysis Based on Clinical Trials. PLoS ONE.

[57] Deng H.-Y., Wang W.-P., Wang Y.-C., Hu W.-P., Ni P.-Z., Lin Y.-D., Chen L.-Q. (2017). Neoadjuvant Chemoradiotherapy or Chemotherapy? A Comprehensive Systematic Review and Meta-Analysis of the Options for Neoadjuvant Therapy for Treating Oesophageal Cancer. Eur. J. Cardio-Thoracic Surg..

[58] Xu A.-M., Huang L., Liu W., Gao S., Han W.-X., Wei Z.-J. (2014). Neoadjuvant Chemotherapy Followed by Surgery versus Surgery Alone for Gastric Carcinoma: Systematic Review and Meta-Analysis of Randomized Controlled Trials. PLoS ONE.

[59] Liao Y., Yang Z., Peng J., Xiang J., Wang J. (2013). Neoadjuvant Chemotherapy for Gastric Cancer: A Meta-Analysis of Randomized, Controlled Trials. J. Gastroenterol. Hepatol..

[60] Fiorica F., Cartei F., Enea M., Licata A., Cabibbo G., Carau B., Liboni A., Ursino S., Cammà C. (2007). The Impact of Radiotherapy on Survival in Resectable Gastric Carcinoma: A Meta-Analysis of Literature Data. Cancer Treat. Rev..

[61] Yang Y., Yin X., Sheng L., Xu S., Dong L., Liu L. (2015). Perioperative Chemotherapy More of a Benefit for Overall Survival than Adjuvant Chemotherapy for Operable Gastric Cancer: An Updated Meta-Analysis. Sci. Rep..

[62] ClinicalTrials.gov. https://clinicaltrials.gov/.

[63] Däster S., Eppenberger-Castori S., Mele V., Schäfer H.M., Schmid L., Weixler B., Soysal S.D., Droeser R.A., Spagnoli G.C., Kettelhack C. (2020). Low Expression of Programmed Death 1 (PD-1), PD-1 Ligand 1 (PD-L1), and Low CD8+ T Lymphocyte Infiltration Identify a Subgroup of Patients with Gastric and Esophageal Adenocarcinoma With Severe Prognosis. Front. Med..

[64] Chen K., Cheng G., Zhang F., Zhang N., Li D., Jin J., Wu J., Ying L., Mao W., Su D. (2016). Prognostic Significance of Programmed Death-1 and Programmed Death-Ligand 1 Expression in Patients with Esophageal Squamous Cell Carcinoma. Oncotarget.

[65] Kollmann D., Ignatova D., Jedamzik J., Chang Y.T., Jomrich G., Baierl A., Kazakov D., Michal M., French L.E., Hoetzenecker W. (2018). PD-L1 Expression Is an Independent Predictor of Favorable Outcome in Patients with Localized Esophageal Adenocarcinoma. Oncoimmunology.

[66] Dislich B., Stein A., Seiler C.A., Kröll D., Berezowska S., Zlobec I., Galvan J., Slotta-Huspenina J., Walch A., Langer R. (2017). Expression Patterns of Programmed Death-Ligand 1 in Esophageal Adenocarcinomas: Comparison between Primary Tumors and Metastases. Cancer Immunol. Immunother..

[67] Bensch F., van der Veen E.L., Lub-de Hooge M.N., Jorritsma-Smit A., Boellaard R., Kok I.C., Oosting S.F., Schröder C.P., Hiltermann T.J.N., van der Wekken A.J. (2018). 89Zr-Atezolizumab Imaging as a Non-Invasive Approach to Assess Clinical Response to PD-L1 Blockade in Cancer. Nat. Med..

[68] Gerson J.N., Skariah S., Denlinger C.S., Astsaturov I. (2017). Perspectives of HER2-Targeting in Gastric and Esophageal Cancer. Expert Opin. Investig. Drugs.

[69] Bensch F., Brouwers A.H., Lub-de Hooge M.N., de Jong J.R., van der Vegt B., Sleijfer S., de Vries E.G.E., Schröder C.P. (2018). 89Zr-Trastuzumab PET Supports Clinical Decision Making in Breast Cancer Patients, When HER2 Status Cannot Be Determined by Standard Work Up. Eur. J. Nucl. Med. Mol. Imaging.

[70] Ai D., Ye J., Wei S., Li Y., Luo H., Cao J., Zhu Z., Zhao W., Lin Q., Yang H. (2022). Comparison of 3 Paclitaxel-Based Chemoradiotherapy Regimens for Patients with Locally Advanced Esophageal Squamous Cell Cancer. JAMA Netw. Open.

[71] Hu Y., Yoon S.S. (2021). Extent of Gastrectomy and Lymphadenectomy for Gastric Adenocarcinoma. Surg. Oncol..

[72] Jones J.O., Smyth E.C. (2020). Gastroesophageal Cancer: Navigating the Immune and Genetic Terrain to Improve Clinical Outcomes. Cancer Treat. Rev..

[73] Joshi S.S., Badgwell B.D. (2021). Current Treatment and Recent Progress in Gastric Cancer. CA Cancer J. Clin..

[74] Pericay C., Macías-Declara I., Arrazubi V., Vilà L., Marín M. (2019). Treatment in Esophagogastric Junction Cancer: Past, Present and Future. Cir. Esp..

